# Curriculum framework for artificial intelligence literacy in veterinary education

**DOI:** 10.3389/fvets.2026.1801756

**Published:** 2026-04-22

**Authors:** Yi-Ting Huang, Candice P. Chu

**Affiliations:** Department of Veterinary Pathobiology, College of Veterinary Medicine & Biomedical Sciences, Texas A&M University, College Station, TX, United States

**Keywords:** AI, AI literacy, artificial intelligence, DVM education, large language model, machine learning, veterinary curriculum, veterinary student

## Abstract

Artificial intelligence (AI) is a rapidly evolving technology increasingly incorporated into veterinary medicine. Given the demand for AI integration and the accountability placed on licensed veterinarians for AI-assisted medical decisions, there is an urgent need to integrate AI literacy into the Doctor of Veterinary Medicine (DVM) curriculum in the United States. Here, we proposed five core modules for a comprehensive AI literacy curriculum for DVM students that can serve as a foundation for institution-specific course development. We also urge institutions to provide the resources outlined in this article to facilitate the development of AI literacy courses in veterinary schools.

## 1 Introduction

Artificial intelligence (AI) is a rapidly evolving technology and is increasingly incorporated into veterinary medicine. AI has already been implemented in image evaluation-based diagnostics, such as detecting uroliths on canine abdominal radiographs and generating cytologic interpretations for lymph nodes and skin masses (1, 2). AI-powered scribe companies have also collaborated with veterinary teaching hospitals, such as Texas A&M University, to improve the efficiency and accuracy of medical records in veterinary practice (3–6). With the inevitability of AI integration into veterinary medicine, the practitioners, as well as the future veterinarians, must be informed of its potential and risks. Several cross-sectional surveys report that veterinary students are more optimistic about integrating AI into clinical practice when they are familiar with the technology (7–11). Despite generally positive attitudes toward AI, most veterinary students describe themselves as only moderately knowledgeable and even less confident in using AI for learning (10). Consequently, many veterinary students express a strong interest in coursework that integrates AI and its appropriate use in future practice (7–10). In parallel, veterinary associations have issued statements encouraging members to develop AI literacy while emphasizing that AI should remain assistive, used under the supervision of a licensed professional (12–20). However, surveys reported that many veterinarians do not use AI in their practices because of unfamiliarity with the technology or concerns about ethics (11, 21). Given the increasing demand for AI integration in veterinary medicine (7–11, 22) and the expectation that licensed veterinarians remain accountable for medical decisions informed by AI output (12–20), there is an urgent need to integrate AI literacy into the Doctor of Veterinary Medicine (DVM) curriculum.

## 2 Review and proposal for AI curriculum framework

### 2.1 AI curriculum frameworks from human medicine relevant to DVM education

In human medicine, many articles and professional associations have called for more comprehensive education on AI use, but no widely accepted curriculum currently exists. Tolentino et al. proposed an AI curriculum framework for medical professionals in a 2024 review, organized into 2 main topics and 9 subcategories (23). The first topic centers on AI and its implications, including core concepts, potential clinical value, limitations, and ethical and legal issues. The second topic emphasizes practical use, including identifying appropriate tasks, integrating AI into workflows, understanding impact, disclosing AI use to patients, and critically assessing AI outputs (23). Similarly, Stanford Medical School's *Artificial Intelligence in Clinical Practice: A Curriculum for Medical Students* describes a peer-reviewed, consensus-developed course that includes foundational knowledge, AI integration skills, ethical and legal considerations, and critical appraisal of AI models (24). This course design aligns with the framework proposed by Tolentino et al. and may inform curriculum development for veterinary students (23, 24).

### 2.2 Current landscape of AI courses offered in the U.S. veterinary schools

Several U.S. veterinary schools currently offer AI-related courses for DVM students. At Auburn University, the course *Spectrum of Care Series: Emerging Technology* includes an introduction to AI and its application (25). At the University of Wisconsin-Madison, the elective course *Artificial Intelligence for Veterinary Medicine* emphasizes building a machine learning tool to recognize patterns in veterinary medicine and validating performance (26). Kansas State University's *Artificial Intelligence in Veterinary Medicine* places greater emphasis on how AI can be used in learning and clinical practice (27). At Texas A&M University, a new AI elective for second-year veterinary students, *Artificial Intelligence and Digital Tools for Next-Generation Veterinarians*, covers topics similar to those in the Kansas State University curriculum, with additional content on prompt engineering, AI for clinical research and scientific writing, and hands-on exercises in computer vision model training and AI chatbot development (28).

### 2.3 Proposed AI literacy curriculum for veterinary students

Here, we propose five core modules for a comprehensive AI literacy curriculum for DVM students that can serve as a foundation for institution-specific course development. The content is informed by the framework proposed by Tolentino et al. (23), the Stanford Medical School curriculum (24), and existing veterinary AI courses. An example weekly schedule is provided in Table 1.

**Table 1:** Example weekly schedule from *VTPB 948 301: artificial intelligence and digital tools for next-generation veterinarians*, a one-credit AI literacy course for veterinary students at Texas A&M University.

Week	Module	Topic	Description
1		Introduction	Overview of course objectives, expectations, assignments, and grading structure.
2	1	Fundamentals of artificial intelligence (AI)	Description of foundational AI concepts, including machine learning, neural networks, and deep learning. This lesson covers the “physiology of AI” to ensure all students are informed on the basics of AI.
3	2	Machine learning (ML)	Summary of major ML paradigms, including model training, validation, and testing. This course is integrated with clinical veterinary examples illustrating data preparation workflows and interpretation of performance metrics to showcase ML's potential and limitations.
4	2	ML exercise: Train your ML model	Hands-on exercise in cytologic cell annotation and supervised deep learning workflows for canine lymphoma classification using AnyLabeling and YOLOv11.
5	3	Large language models (LLM) and prompt engineering	Explanation on the basics of LLMs, including transformer-based architectures, and principles of effective prompt engineering for veterinary applications. Characteristics of an effective prompt are discussed and tested.
6	3	LLM exercise: build your AI chatbot	Hands-on exercise in developing a task-specific AI chatbot using structured prompt engineering techniques.
7	3	LLM in veterinary clinical research	Introduction to the principles of natural language processing in veterinary clinical research, with a practical focus on extracting structured data from unstructured clinical narratives and selecting appropriate AI tools based on dataset size, accuracy needs, and privacy considerations.
8	4	Landscape of current veterinary AI products	Examination of current AI-driven products in veterinary medicine, including applications in notetaking, radiology, clinical pathology, and patient monitoring. A clear view of available commercial products gives future veterinarians prepares them for real-world practice.
9	4	Overview of veterinary organizations' position statements on AI	Overview of position and policy statements on AI published by major veterinary professional organizations. This gives students a clear idea of the standard of ethical practice provided by organizations of authority. Additionally, an exercise on appropriate questions to ask AI product vendors before purchasing was designed based on policy statement recommendations.
10	4	Legal considerations and liability when using AI tools in clinics	Discussion of client informed consent, liability considerations, and relevant legal scenarios related to the clinical use of AI tools. Discussion is facilitated by an attorney at law that is familiar with AI related policies.
11	5	AI in academic literature search	Comparison of traditional and AI-enhanced literature search strategies in veterinary medicine, demonstrating how Google Scholar, PubMed, and generative AI tools can be combined to efficiently identify clinically relevant evidence.
12	5	Ethical use of AI in scientific writing	Discussion of ethical considerations surrounding AI-assisted scientific writing, including current regulations, best practices, and potential risks.
13	5	AI and digital tools for slide presentation	Practical session on preparing scientific presentations using AI-assisted and digital tools such as NotebookLM, Research Bites, Gamma, and Claude for PowerPoint (44–47).
14–15		Final project presentations	Student presentations demonstrating application of AI tools to veterinary problems or proposing novel AI-based solutions. Students may take this opportunity to envision the form of AI they would like to see in clinical practice, discuss with the instructor, and share amongst peers.

#### 2.3.1 Module 1: fundamentals of AI

Adopting any new tool requires understanding its core principles. While veterinarians are not expected to master the mathematical or algorithmic details of AI models, familiarity with foundational concepts supports responsible use and appropriate skepticism. An introductory session should define AI, machine learning (ML), deep learning (DL), and major model families (e.g., neural networks, convolutional neural networks [CNNs], and large language models [LLMs]). This content has been described as “the physiology of AI” by Dr. Dong-han Yao at Stanford Medicine as a relatable anecdote for those involved in the medical field (24). Instructors may refer to existing veterinary review articles for additional background (29, 30). At the end of the course, students should be able to explain the basic concept of AI in layman's terms.

#### 2.3.2 Module 2: machine learning

Machine learning (ML) underpins many commercial AI products in veterinary cytology, pathology, and radiology. Professional organizations commonly emphasize that veterinarians should understand ML model development (training, validation, and testing) and key performance metrics to evaluate product strengths and limitations (15, 16). Upon completion of this module, students should be able to interpret basic performance metrics, identify common pitfalls in model evaluation, and critically appraise claims made in commercial AI product marketing. Instructors may use peer-reviewed research articles as examples to guide students through model development and evaluation. An example is provided here (2). An exercise can be adapted from the *Pathology Informatics Workshop: Automated Image Analysis Using Machine Learning*, delivered at the 2025 American College of Veterinary Pathologists (ACVP) Annual Meeting (31).

#### 2.3.3 Module 3: large language model (LLM) and prompt engineering

Large language models (LLMs), such as ChatGPT, Gemini, Grok, and Claude, can generate variable outputs across prompts and sessions. Understanding LLM fundamentals and prompt engineering principles can improve reliability and help users interpret model limitations (30). Creating a clear and efficient prompt is also crucial for achieving consistent, accurate results with LLMs. This module introduces how LLMs can support clinical research tasks, such as data extraction from veterinary records. By the end of this module, students should be able to construct structured prompts for veterinary clinical and research tasks and critically evaluate LLM-generated outputs for accuracy and hallucinations. A suggested paired activity involves building an AI chatbot using an LLM application programming interface (API). Class activities can be developed based on official LLM tutorials to learn the most effective way to implement AI into veterinary practice (32). Additionally, instructors can incorporate newer concepts such as AI agents, vibe coding, along with trending tools like Claude Code, OpenClaw, and Google Stitch (33–37).

#### 2.3.4 Module 4: ethical and legal considerations of AI use

This module focuses on the ethics of using AI in veterinary practice and the veterinarians' responsibility for implementing such technologies. This module first provides an overview of AI tools currently available in the veterinary market, including applications for note-taking, diagnostic imaging, cytology and pathology, clinical decision support, and monitoring (16). Then, current guidelines and legal expectations of veterinarians will be discussed. In North America, AI products used in veterinary medicine currently lack U.S. Food and Drug Administration (FDA) premarket requirements. Veterinary professional organizations have issued position statements encouraging AI adoption aligned with expert consensus and professional standards (12–20). Across these statements, a consistent principle is that AI is assistive: veterinarians remain responsible for final decisions and for communicating relevant information to clients (12–20). While no current legislative regulations are in effect, veterinarians are still expected to act in accordance with ethical practice as required by each state board. These expectations should be explicitly addressed with veterinary students. Upon completion of this module, students should be able to articulate the veterinarian's legal and ethical responsibilities when integrating AI-assisted outputs into clinical decision-making.

#### 2.3.5 Module 5: AI in veterinary scientific research

This module equips DVM students with skills to locate, organize, and synthesize scientific literature using AI-enabled tools. These competencies are increasingly important given the rapid growth in veterinary literature. Examples of retracted articles can be used to illustrate ethical and responsible AI use in scientific writing and presentations, including transparency, authorship integrity, and compliance with journal and institutional policies (30). Additionally, AI's language-processing capabilities have opened the door to large-scale extraction and review of medical records from various databases (38). This content is particularly relevant to some veterinary specialties in which scientific publication is required for board certification (39). By the end of this module, students should be able to use AI-enabled tools to efficiently locate and synthesize scientific literature, compare AI-assisted and traditional search strategies, and identify examples of inappropriate AI use in published work to uphold standards of authorship integrity and transparency.

## 3 AI educational resources for veterinary educators

### 3.1 Who should be teaching AI to veterinary students?

Although AI is traditionally situated within computer science and engineering, many AI courses in human and veterinary medicine are taught by clinicians. This pattern appears consistent across current AI courses offered in U.S. veterinary schools (25–28). At the Stanford AI in Medical Education Symposium in June 2025, most speakers were physicians with an interest in AI, while an attorney also contributed perspectives on ethical and legal implications (24). Because AI capabilities are evolving faster than many regulations and guidelines, legal questions may remain unsettled and context-dependent (24). Input from legal professionals may therefore be of significant value. Instructors could consider inviting guest lecturers (e.g., attorneys, computer scientists, and librarians) to support relevant AI topics.

### 3.2 Institutional support in veterinary AI education

Institutional resources (tools, policies, and training) are essential for scalable AI education. First, institutions should provide access to approved AI tools that align with their institutional data classification requirements (40). Second, universities should establish clear guidance for educators, including syllabus language that defines acceptable AI use in coursework (41). Third, faculty development is necessary. For example, Texas A&M University maintains a “Teach with AI” resource that provides faculty with use cases, assignment ideas, and learning opportunities (42). The Texas A&M Library also offers ready-to-use AI literacy modules on generative AI, such as the use of LLMs in academic research, and ethical considerations (43). A realistic understanding of AI depends on educators' familiarity with its capabilities and limitations. Faculty should recognize that AI detection tools are imperfect, and suspected inappropriate use should be evaluated through additional inquiry (10).

## 4 Conclusion

We proposed an AI literacy curriculum comprising five core modules and provided an example syllabus for veterinary education. We also highlighted readings, tutorials, and ready-to-use course components to support adoption. We anticipate that this framework will expand AI literacy course offerings within veterinary curricula.

## Data Availability

The original contributions presented in the study are included in the article/supplementary material, further inquiries can be directed to the corresponding author.
